# Advances in Solar Cell Materials and Structures—Second Edition

**DOI:** 10.3390/ma19143056

**Published:** 2026-07-16

**Authors:** Grzegorz Wisz, Mirosław Łabuz, Lyubomyr Nykyruy, Rostyslav Yavorskyi

**Affiliations:** 1 Institute of Materials Engineering, University of Rzeszów, 35-310 Rzeszów, Poland; 2 Institute of Physics, University of Rzeszów, 35-310 Rzeszów, Poland; mlabuz@ur.edu.pl; 3 Physics and Chemistry of Solids Department, Physical-Technical Faculty, Vasyl Stefanyk Carpathian National University, 76018 Ivano-Frankivsk, Ukraine; lyubomyr.nykyruy@pnu.edu.ua (L.N.);

## 1. Introduction and Special Issue Scope

The theme of this Special Issue, entitled “Advances in Solar Cell Materials and Structures”, directly addresses the key challenges facing contemporary renewable energy engineering in the context of the global energy crisis and the urgent need to reduce dependence on fossil fuels [Contribution 1]. Despite the market dominance of conventional silicon-based photovoltaic technologies, thin-film solar cells represent a highly efficient and economically attractive alternative. They are characterised by absorber layers that are up to 200 times thinner, lower consumption of raw materials, and the ability to be deposited onto flexible substrates, enabling their integration into systems with diverse geometries [Contribution 1]. As emphasised in recent review studies, thin-film technologies enable the fabrication of lightweight and flexible photovoltaic modules while significantly reducing the amount of active material required, making them one of the most promising classes of solutions for future energy applications [Contribution 2].

The papers collected in this Special Issue address existing knowledge gaps related to the optimisation of the optical, electronic, and structural properties of advanced photovoltaic devices. The scope of the Issue encompasses both advanced mathematical and physical modelling approaches as well as experimental fabrication and characterisation techniques applied to perovskite, dye-sensitised, organic, and conventional thin-film solar cells. The primary motivation behind this collection was to bring together recent advances in defect engineering, interface modification, nanoscale phase separation control, and optimisation of transparent conductive oxide (TCO) layers. These contributions not only consolidate the current state of knowledge but also identify emerging research directions aimed at enhancing long-term stability, increasing power conversion efficiency, and reducing the manufacturing costs of next-generation photovoltaic devices. These directions are closely aligned with current trends in thin-film photovoltaics, where particular emphasis is placed on improving operational durability, optimising multijunction architectures, and facilitating the industrial implementation of novel functional materials [Contribution 3]. At the same time, the published studies highlight the increasing integration of materials research, advanced numerical modelling, interface engineering, and monitoring systems for photovoltaic devices. This multidisciplinary approach is currently becoming one of the key driving forces behind the development of next-generation photovoltaic technologies aimed not only at maximising energy conversion efficiency but also at ensuring long-term operational stability and enabling industrial-scale implementation.

## 2. An Overview of the Special Issue

The opening review article by Salgado-Conrado and co-workers presents a critical analysis of thirteen leading numerical simulation tools, including SCAPS, AMPS, AFORS-HET, GPVDM, SILVACO, and SENTAURUS, widely used for modelling and optimising photovoltaic device architectures [Contribution 1]. The authors provide a comprehensive comparison of computational algorithms, charge carrier transport models, and licencing frameworks, demonstrating that the appropriate choice of simulation software enables accurate prediction of device performance while reducing the need for costly and time-intensive experimental investigations. The findings of this review highlight the growing importance of computational approaches as an integral component of advanced photovoltaic device design and highlight the ongoing transition from conventional trial-and-error experimental methodologies to research strategies supported by numerical modelling.

In the second research article, Wisz and co-workers investigated the properties of TiO_2_/Cu_x_O oxide heterojunctions deposited by reactive direct-current magnetron sputtering (DC-MS) onto indium tin oxide (ITO)-coated glass substrates [Contribution 2]. The study examined the influence of post-deposition annealing in air at 150 °C on the optical and structural properties of the heterojunctions. The experimental results revealed that thermal treatment induced favourable modifications in the surface morphology and optical characteristics of the deposited layers, which translated into improved current–voltage (Contributions 1–5) characteristics and enhanced power conversion efficiency of the annealed structures. These findings confirm the crucial role of post-deposition processes in tailoring the functional properties of oxide heterojunctions and indicate that properly selected annealing conditions can serve as an effective strategy for optimising the performance of thin-film solar cells.

The subsequent contribution by Amin and co-workers focuses on dye-sensitised solar cells (DSSCs) and reports the synthesis of three novel donor–acceptor (D–A) organic dyes based on a phenothiazine core, employing 1H-tetrazolo-5-acrylic acid as both the anchoring group and electron-accepting moiety [Contribution 3]. Electrochemical and calorimetric analyses revealed that these compounds exhibit molecular glass behaviour and possess HOMO and LUMO energy levels that are favourably aligned with the conduction band of TiO_2_ and the redox potential of the electrolyte. To enhance photoanode performance, the authors combined co-sensitisation and co-adsorption strategies with the incorporation of an additional blocking layer. This approach resulted in a substantial improvement in device performance, yielding a power conversion efficiency (PCE) of 6.37%. The study demonstrates that further advancement of DSSCs relies not only on the design of novel chromophoric systems but also on the effective control of interfacial charge-transfer processes and optimisation of photoanode architectures.

In the fourth paper, Guzowski and co-workers proposed an approach for photovoltaic system diagnostics by developing a fibre-optic temperature sensor based on a Mach–Zehnder interferometer [Contribution 4]. The sensing structure, fabricated by introducing tapered regions into a single-mode SMF-28e+ optical fibre, was coated with a 100 nm-thick gold layer to assess its effect on thermal sensitivity. Spectral characterisation carried out in the O, S, C, and L bands over the temperature range of 0–70 °C revealed that the metallic coating enabled a maximum temperature sensitivity of up to 72 pm/°C while maintaining low average transmission losses below 7 dB. These characteristics make such structures promising candidates for condition-monitoring systems of photovoltaic installations operating under real-world conditions. The study broadens the conventional scope of photovoltaic research by incorporating operational diagnostics and is consistent with current trends toward intelligent monitoring systems that allow a predictive assessment of the technical condition of energy installations.

The fifth contribution, by Khan and co-workers, is a critical assessment of the use of solid additives in bulk heterojunction (BHJ)-based organic solar cells (OSCs). The authors classify these components into non-volatile additives, volatile additives, and nanomaterials, and discuss in detail their roles in optimising device morphology and performance [Contribution 5]. The review consolidates current knowledge of the mechanisms governing nanoscale phase separation during thin-film formation and examines how the molecular architecture of additives influences key photovoltaic parameters, including the open-circuit voltage (V_oc_), short-circuit current density (J_sc_), fill factor (FF), and the long-term thermal and operational stability of the active layer. The analysis demonstrates that the rational design of functional additives remains one of the most effective strategies for enhancing both the efficiency and durability of organic solar cells, particularly through precise control of the active-layer morphology at the nanoscale.

In the final article, Mientki and co-workers developed a novel physical vapour co-deposition (PVco-D) approach combined with post-deposition annealing for the fabrication of copper-doped zinc oxide (ZnO:Cu) thin films as a cost-effective and environmentally friendly alternative to conventional ITO electrodes [Contribution 6]. Structural characterisation using atomic force microscopy (AFM), X-ray fluorescence (XRF) analysis, and electrical measurements confirmed the high transparency, structural uniformity, and ohmic conduction behaviour of the fabricated films. On the basis of the experimentally determined electrical properties, the authors incorporated the optimised ZnO:Cu material into a numerical model of a thin-film perovskite solar cell, demonstrating the potential to achieve a theoretical power conversion efficiency of approximately 17%. These findings highlight the potential of ZnO-based materials as promising alternatives to conventional indium-containing transparent conductive oxide (TCO) electrodes, which is particularly significant in the context of sustainable development and efforts to reduce dependence on critical raw materials.

## 3. Conclusions

This editorial provides an overview of six scientific contributions that present recent advances in photovoltaic technologies, explore the mechanisms and factors governing energy conversion efficiency, and examine emerging trends in numerical modelling and diagnostic methodologies for solar cells. The studies collected in this Special Issue provide valuable insight for engineers, researchers, and technologists working in the fields of advanced thin-film materials, surface engineering, and characterisation techniques. Collectively, these contributions highlight the growing prospects of solar technologies, which are closely associated with increasing automation and digitalisation of design processes, the implementation of intelligent monitoring systems, and the search for materials and methodologies capable of improving energy efficiency while minimising the environmental impact of manufacturing processes.

The critical evaluation of computational tools demonstrated that simplified one-dimensional (1D) simulators, such as SCAPS and AMPS [Contribution 1] [1,2], while highly effective for modelling fundamental charge transport mechanisms, have inherent limitations in describing spatially non-uniform geometries. Consequently, the accurate representation of two- and three-dimensional (2D/3D) effects requires the use of more advanced simulation platforms, such as Silvaco ATLAS and Sentaurus TCAD [Contribution 1]. From the perspective of materials engineering, one of the most significant application-oriented findings presented in this Special Issue is that physical vapour co-deposition (PVco-D) of ZnO:Cu layers offers a viable route toward replacing conventional ITO electrodes while maintaining ohmic conduction behaviour and enabling a theoretical power conversion efficiency of approximately 17% in perovskite solar cells [Contribution 6]. Experimental investigations of TiO_2_/Cu_x_O heterostructures and organic photovoltaic systems clearly demonstrated that operational stability, optoelectronic performance, and layer morphology are strongly influenced by controlled post-deposition annealing treatment in air [Contribution 2] [3], molecular engineering of sensitiser architectures [Contribution 3], and the rational design of non-volatile and nanomaterial-based solid additives [Contribution 5]. The synergy of these approaches, complemented by the implementation of Mach–Zehnder interferometric fibre-optic sensors with a temperature sensitivity of up to 72 pm/°C, extends the photovoltaic design framework by enabling accurate thermal diagnostics of photovoltaic modules under real operating conditions [Contribution 4].

These findings underscore the importance of an interdisciplinary approach that integrates materials engineering, numerical modelling, fabrication technologies, and advanced diagnostic systems in the development of next-generation photovoltaic devices. Despite the remarkable progress achieved in recent years, further advancement of photovoltaic technologies will depend on overcoming persistent challenges related to the long-term stability of functional materials and the degradation of active layers during operation, which remain significant barriers to the commercialisation of many emerging photovoltaic technologies [4]. In addition, this analysis highlights the crucial role of collaboration between academia and industry in facilitating knowledge transfer and accelerating the implementation of innovative technologies into engineering practice.

The analysis of the contributions published in this Special Issue reveals a clear shift in the contemporary research paradigm of solar cell technologies. While earlier research was primarily focused on improving power conversion efficiency through the development of novel absorber materials, current efforts increasingly embrace an integrated approach that simultaneously addresses materials design, device architecture, fabrication processes, and operational monitoring. This evolution reflects a growing recognition that achieving high-performance photovoltaic systems requires the coordinated optimisation of all stages of device development and operation [5,6,7,8,9,10,11,12].

Looking ahead, particular attention should be directed toward improving the long-term operational stability of next-generation photovoltaic devices, advancing interface engineering strategies to mitigate charge-carrier recombination losses, applying artificial intelligence and machine learning techniques to the design and optimisation of functional materials, and developing intelligent diagnostic systems capable of monitoring degradation processes in real time. In addition, research should focus on enhancing device durability, suppressing interfacial degradation phenomena, and establishing scalable fabrication technologies that enable the effective transfer of innovative solutions from laboratory-scale demonstrations to industrial production. Collectively, these research directions are expected to play a pivotal role in the continued development of efficient, durable, and economically competitive photovoltaic technologies.
